# Predictive value of CHA_2_DS_2_‐VASc score for in‐hospital prognosis of patients with acute ST‐segment elevation myocardial infarction undergoing primary PCI

**DOI:** 10.1002/clc.24071

**Published:** 2023-07-10

**Authors:** Ying Sun, Jian Ren, Wei Wang, Chunsong Wang, Li Li, Hengchen Yao

**Affiliations:** ^1^ Department of Cardiology, Liaocheng People's Hospital Shandong University Jinan Shandong P.R. China; ^2^ Department of Cardiology Liaocheng People's Hospital Affiliated to Shandong First Medical University Liaocheng Shandong P.R. China; ^3^ Department of Cardiology, Liaocheng Dongchangfu People's Hospital Liaocheng People's Hospital Liaocheng Shandong P.R. China

**Keywords:** acute myocardial infarction, CHA_2_DS_2_‐VASc score, gender difference, major adverse cardiac events, ST‐elevation myocardial infarction

## Abstract

**Background:**

This study aimed to explore the predictive value of CHA_2_DS_2_‐VASc score for in‐hospital major adverse cardiac events (MACEs) in ST‐elevation myocardial infarction (STEMI) patients undergoing primary percutaneous coronary artery intervention.

**Methods:**

A total of 746 STEMI patients were divided into four groups according to CHA_2_DS_2_‐VASc score (1, 2–3, 4–5, >5). The predictive ability of the CHA_2_DS_2_‐VASc score for in‐hospital MACE was made. Subgroup analysis was made between gender differences.

**Results:**

In a multivariate logistic regression analysis model including creatinine, total cholesterol, and left ventricular ejection fraction, CHA_2_DS_2_‐VASc score was an independent predictor of MACE as a continuous variable (adjusted odds ratio: 1.43, 95% confidence interval [CI]: 1.27–1.62, *p* < .001). As a category variable, using the lowest CHA_2_DS_2_‐VASc score of 1 as a reference, CHA_2_DS_2_‐VASc score 2–3, 4–5, >5 groups for predicting MACE was 4.62 (95% CI: 1.94–11.00, *p* = .001), 7.74 (95% CI: 3.18–18.89, *p* < .001), and 11.71 (95% CI: 4.14–33.15, *p* < .001). The CHA_2_DS_2_‐VASc score was also an independent risk factor for MACE in the male group, either as a continuous variable or category variable. However, CHA_2_DS_2_‐VASc score was not a predictor of MACE in the female group. The area under the curve value of the CHA_2_DS_2_‐VASc score for predicting MACE was 0.661 in total patients (74.1% sensitivity and 50.4% specificity [*p* < .001]), 0.714 in the male group (69.4% sensitivity and 63.1% specificity [*p* < .001]), but there was no statistical significance in the female group.

**Conclusions:**

CHA_2_DS_2_‐VASc score could be considered as a potential predictor of in‐hospital MACE with STEMI, especially in males.

## Graphical abstract

A total of 746 patients with ST‐elevation myocardial infarction undergoing primary percutaneous coronary intervention were studied, including 143 patients of major adverse cardiac events (MACEs). Logistic regression analysis showed that CHA_2_DS_2_‐VASc score was an independent predictor of MACE. CHA_2_DS_2_‐VASc score was an independent risk factor for MACE in the male group, but not in the female group.

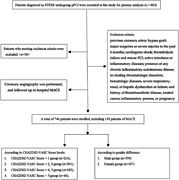

## 1 INTRODUCTION

Acute myocardial infarction (AMI), especially ST‐elevating myocardial infarction (STEMI), caused by occlusion of the coronary artery,^1^ is a fetal disease that represents a major cause of worldwide mortality. The mortality of STEMI has been greatly reduced by the primary percutaneous coronary intervention (pPCI).^2^ However, the incidence of major adverse cardiac events (MACE) including heart failure (HF), cardiac rupture, reinfarction, arrhythmia, angina, and death is very high during the in‐hospital stage of STEMI patients.^3^ So, there is an urgent need to take early action to find predictors of these complications of STEMI and to reduce mortality.

CHA_2_DS_2_‐VASc score is a clinical decision rule developed by Lip et al.,^4^ which is clinically used to assess the risk of thromboembolism in atrial fibrillation (AF) patients and guide anticoagulation treatment.^5^ This scoring system consists of several factors including HF, hypertension, age, diabetes mellitus (DM), previous stroke or transient ischemic attack, vascular disease, and female gender.^4^ In addition to evaluating the thromboembolism risk of nonvalvular AF, it was also proven to be a risk factor for adverse clinical outcomes in stable coronary artery disease (CAD),^6^ acute coronary syndrome (ACS),^7^ including STEMI^8^ and non‐ST‐elevation myocardial infarction (NSTEMI).^9^

However, the prognostic value of the CHA_2_DS_2_‐VASc score for MACE in STEMI patients who underwent pPCI remains unclear. Thus, this study aimed to investigate the predictive value of preprocedural CHA_2_DS_2_‐VASc score for in‐hospital MACE in all subjects, and in subgroups with gender differences.

## 2 METHODS

### 2.1 Study population

In the period from July 2015 to August 2019, consecutive patients with a diagnosis of STEMI admitted into Liaocheng People's Hospital were enrolled in this retrospective study. All patients underwent pPCI within 24 hours after the onset of symptoms. STEMI was diagnosed according to the European Society of Cardiology Guidelines^10^: a chief complaint of continuous typical chest pain for at least 30 minutes and new persistent ST‐segment elevation for at least 1 mm in two contiguous electrocardiography leads within 12 hours of symptom onset or for up to 24 hours if there was evidence of persistent ischemia or hemodynamic instability, or new left bundle‐branch block in the electrocardiogram, and elevation of cardiac biomarkers, including creatine kinase‐MB and troponin I, above the 99th percentile upper reference limit. The diagnosis was confirmed by coronary angiography in all patients. According to hospital records, baseline characteristics and past medical history including hypertension, DM, smoking status, and family history of CAD were collected. Patients with previous coronary artery bypass graft (CABG); major surgeries or severe injuries in the past 6 months; cardiogenic shock; thrombolysis failure and rescue PCI; active infectious or inflammatory diseases; the presence of any chronic inflammatory‐autoimmune disease including rheumatologic disorders, hematologic diseases, severe respiratory, renal, or hepatic dysfunction or failure; and STEMI history of thromboembolic disease, treated cancer, inflammatory process, or pregnancy were excluded from our study.

This study was approved by the Medical Ethics Committee of Liaocheng People's Hospital. All procedures were in accordance with the principles of the Helsinki Declaration.

### 2.2 Blood sample collecting and laboratory testing

Venous blood samples were obtained from patients by standard venipuncture techniques on admission before the pPCI procedure. Laboratory tests were performed by the emergency laboratory of our hospital. Biochemical analysis was performed to measure serum total cholesterol (TC), triglyceride, creatinine, d‐dimer, and blood glucose.

### 2.3 The assessment of CHA_2_DS_2_‐VASc score

On admission, patients were assigned points for HF (1 point), hypertension (1 point), age above 75 years (2 points), DM (1 point) and prior stroke (2 points), age above 65 (1 point), female sex (1 point), and vascular disease (1 point).

### 2.4 The definition of MACE

The endpoints of the study were MACE, including death, revascularization, angina, reinfarction, and new‐onset HF. From enrollment to discharge, MACE was recorded. Death was determined as all‐cause death. Coronary revascularization was confirmed by either PCI or CABG surgery during the in‐hospital period. Angina or reinfarction was diagnosed through ischemic symptoms and electrocardiographic changes with or without elevated serum cardiac enzyme levels. New‐onset HF was determined by clinical signs and symptoms in a physical examination and on cardiac ultrasound and chest radiography.

### 2.5 Statistical analysis

Statistical analysis was carried out using SPSS software 23.0 (IBM Corp.). Shapiro–Wilk test was used to determine whether continuous data were normally distributed or not. Normally distributed numerical variables were expressed as mean ± standard deviation, while non‐normally distributed data were expressed as median (interquartile range). Categorical variables were reported in frequency (percentages). *χ* ^2^ test and Mann–Whitney *U* test were used for the comparisons of categorical and continuous variables, respectively. Independent factors for predicting the incidence of MACE were calculated by univariate logistic analysis, variables with a *p* < .05 in univariate analysis were included in multivariate logistic regression models, and adjusted odds ratios (AOR) were calculated. A receiver operating characteristic (ROC) analysis was made by MedCalc statistical software to further explore the applicability of CHA_2_DS_2_‐VASc score as a potential biomarker in the prediction of MACE. The difference of area under the curve value (AUC) between CHA_2_DS_2_‐VASc score and left ventricular ejection fraction (LVEF), also creatinine for the prediction of MACE was made by *Z* test using MedCalc statistical software. All analyses were two‐sided and *p* < .05 were considered statistically significant.

## 3 RESULTS

### 3.1 Patients characteristics

**Figure 1:**
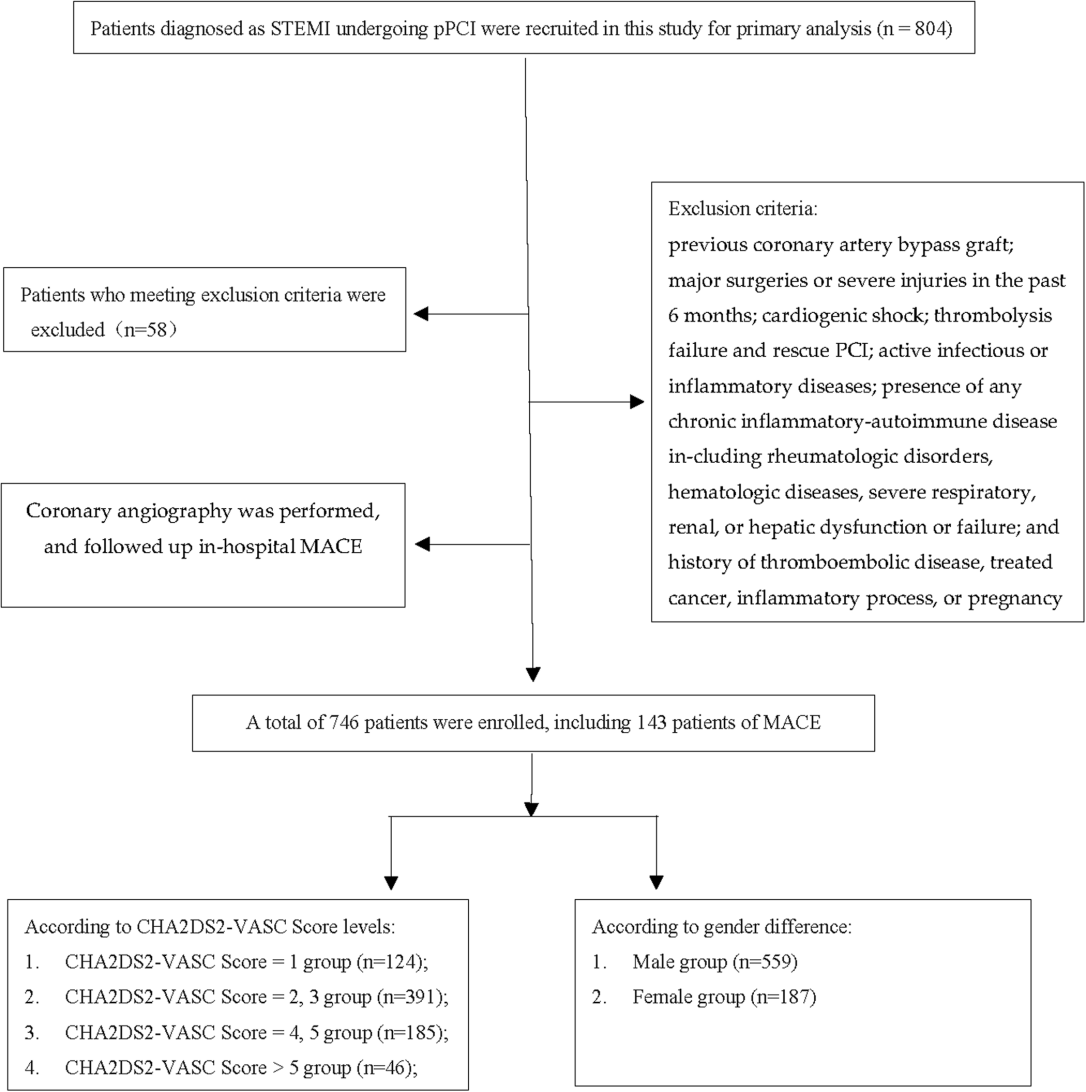
The flowchart of the study. MACE, major adverse cardiac events; pPCI, primary percutaneous coronary intervention; STEMI, ST‐elevation myocardial infarction.

A total of 746 STEMI patients who underwent pPCI were enrolled in this study, including 559 male patients and 187 female patients, the flowchart was shown in Figure 1. According to CHA_2_DS_2_‐VASc score levels, patients were divided into four groups (1, 2–3, 4–5, >5). The difference in age, smoking, DM, hypertension, family history, gender, time, hemoglobin, white blood cells(WBC), fibrinogen (Fib), and d‐dimer among the four groups was statistically significant and detailed in Table 1. Also, we divided the patients into two groups according to CHA_2_DS_2_‐VASc score levels (low <2, high ≥2). As shown in Supporting Information: Table 1, the difference in age, smoking, DM, hypertension, family history, gender, hemoglobin, WBC, creatinine, and Fib between the two groups was also statistically significant, respectively. Subgroup analysis was made according to gender difference, the basic characteristics are shown in Supporting Information: Tables 2 and 3.

**Table 1:** Basic characteristics of patients with CHA_2_DS_2_‐VASc score difference.

Characteristics	1, *n* = 124	2–3, *n* = 391	4–5, *n* = 185	>5, *n* = 46	*p* Value
Age (year)	51 (14)	60 (16)	68 (11)	73 (10)	<.001
Smoking, *n* (%)	92 (74.2)	221 (56.5)	76 (41.1)	16 (34.8)	<.001
DM, *n* (%)	0 (0)	100 (25.6)	69 (37.3)	29 (63.0)	<.001
Hypertension, *n* (%)	0 (0)	231 (59.1)	127 (68.6)	38 (82.6)	<.001
Family history, *n* (%)	4 (3.2)	42 (10.7)	27 (14.6)	11 (23.9)	<.001
Past CAD, *n* (%)	11 (8.9)	30 (7.7)	11 (5.9)	0 (0)	.192
Male, *n* (%)	124 (100)	313 (80.1)	102 (55.1)	20 (43.5)	<.001
Time (h)	3 (4)	4 (4)	4 (3.75)	5.25 (4.13)	<.05
Heart rate (bpm)	78.3 ± 17.10	76 (20)	75 (21)	78.04 ± 16.63	.222
Hemoglobin (g/L)	149 (14)	146 (21)	136.28 ± 17.35	132.7 ± 12.2	<.001
WBC count (×10^9^/L)	10.29 (3.97)	9.72 (4.06)	9.29 (3.67)	10.29 ± 4.81	<.05
NEU (×10^9^/L)	8.0 (3.88)	7.54 (4.28)	7.23 (3.87))	8.32 ± 3.51	.184
PLT (×10^9^/L)	234 (78)	231 (80)	218 (70)	234 (68)	.189
LYM (×10^9^/L)	1.40 (1.10)	1.34 (1.00)	1.32 (0.92)	1.31 (0.73)	.554
Creatinine (µmol/L)	66 (17)	61.90 (21)	63 (21)	60.80 (28)	.186
TC (mmol/L)	4.80 (1.18)	4.66 (1.25)	4.68 (1.39)	4.86 ± 1.57	.616
TG (mmol/L)	1.49 (1.18)	1.46 (1.26)	1.46 (1.19)	1.55 (1.16)	.869
LVEF (%)	50.52 ± 7.04	50 (10)	50 (10)	51.61 ± 6.93	.677
Fib (ng/mL)	2.92 (0.75)	2.98 (0.93)	3.05 (0.84)	3.10 ± 0.74	<.05
d‐dimer (ng/mL)	0.30 (0.41)	0.30 (0.44)	0.40 (0.59)	0.47 (0.66)	<.001
N/L	6.17 (6.18)	5.70 (5.6)	5.93 (5.41)	6.05 (6.24)	.789

Abbreviations: CAD, coronary artery disease; DM, diabetes mellitus; Fib, fibrinogen; LVEF, left ventricular ejection fraction; LYM, lymphocyte; NEU, neutrophils; N/L, neutrophils‐to‐lymphocyte ratio; PLT, platelet; TC, total cholesterol; TG, triglyceride; WBC, white blood cell.

### 3.2 Clinical outcomes of adverse cardiovascular events

In‐hospital MACE was calculated among all the patients. Incidence of MACE was 19.2% (143 out of 746 patients), including instances of cardiovascular death (*n* = 14), angina (*n* = 49), revascularization (*n* = 3), reinfarction (*n* = 4), and new onset of HF (*n* = 73). The total MACE rate in the high CHA_2_DS_2_‐VASc score group was higher compared with that of the low group (22.0% vs. 4.8%, *p* < .001). Furthermore, the incidence of angina and new‐onset HF between the two groups was statistically significant (*p* < .05 and *p* = .001, respectively). However, there was no difference in the incidence of death, revascularization, or reinfarction (as shown in Table 2).

**Table 2:** Major adverse cardiac events according to CHA_2_DS_2_‐VASc score difference.

Complications	CHA_2_DS_2_‐VASc score category		*p* Value
	Low < 2, *n* = 124	High ≥ 2, *n* = 622	
Death, *n* (%)	1 (0.8)	13 (2.1)	.336
Angina, *n* (%)	3 (2.4)	46 (7.4)	<.05
Revascularization, *n* (%)	0 (0)	3 (0.5)	.438
Reinfarction, *n* (%)	0 (0)	4 (0.6)	.371
New onset heart failure, *n* (%)	2 (1.6)	71 (11.4)	.001
Total MACE, *n* (%)	6 (4.8)	137 (22.0)	<.001

Abbreviation: MACE, major adverse cardiovascular event.

### 3.3 Logistic regression analysis for prediction of MACE

In the multivariate logistic regression analysis model including creatinine, TC, and LVEF, the CHA_2_DS_2_‐VASc score was an independent predictor of MACE as a continuous variable (AOR: 1.43, 95% confidence interval [CI]: 1.27–1.62, *p* < .001). As a category variable, using the lowest CHA_2_DS_2_‐VASc score of 1 as a reference, CHA_2_DS_2_‐VASc score 2–3, 4–5, >5 groups for predicting MACE was 4.62 (95% CI: 1.94–11.00, *p* = .001), 7.74（95% CI: 3.18–18.89, *p* < .001), and 11.71 (95% CI: 4.14–33.15, *p* < .001) (Table 3).

**Table 3:** Logistic regression analysis to show MACE predicted by CHA_2_DS_2_‐VASc score in all patients.

Scoring algorithm	Univariable analysis			Multivariable analysis		
	OR	95% CI	*p*	AOR	95% CI	*p*
CHA_2_DS_2_‐VASc (continuous variable)^a^	1.41	1.25–1.58	<.001	1.43	1.27–1.62	<.001
CHA_2_DS_2_‐VASc (category variable)^b^						
1	Reference	–	–	Reference	–	–
2–3	4.36	1.85–10.31	.001	4.62	1.94–11.00	.001
4–5	7.28	3.02–17.60	<.001	7.74	3.18–18.89	<.001
> 5	10.49	3.78–29.10	<.001	11.71	4.14–33.15	<.001

Abbreviation: AOR, adjusted odds ratio; CI, confidence interval; MACE, major adverse cardiovascular event; OR, odds ratio.

^a^ The multivariable analysis model included the CHA_2_DS_2_‐VASc score as a continuous variable, creatinine, total cholesterol and left ventricular ejection fraction.

^b^ The multivariable analysis model included the CHA_2_DS_2_‐VASc score as a category variable, creatinine, total cholesterol and left ventricular ejection fraction.

Subgroup analysis was made according to gender difference, CHA_2_DS_2_‐VASc score as a continuous variable or category variable was included in the logistic analysis, respectively. The CHA_2_DS_2_‐VASc score was also an independent risk factor for MACE in the male group, either as a continuous variable or a category variable. However, the CHA_2_DS_2_‐VASc score was not a predictor of MACE in the female group. As shown in Supporting Information: Tables 4 and 5. Interestingly, creatinine and LVEF were predictors for MACE in both male and female groups.

### 3.4 ROC curve to show the predictive value of CHA_2_DS_2_‐VASc score for in‐hospital MACE

**Figure 2:**
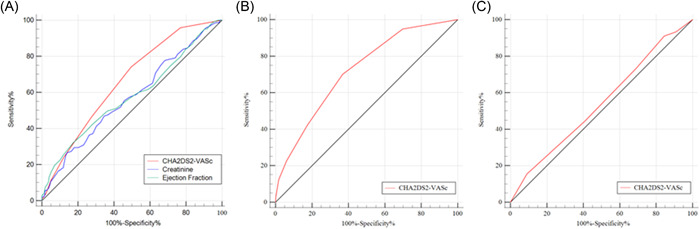
Performance of variables in predicting short‐term major adverse cardiac events. (A) Receiver operating characteristics curve (ROC) of CHA2DS2‐VASc score, creatinine and ejection fraction for the prediction of MACEs. (B) ROC of CHA2DS2‐VASc score for the prediction of MACEs in the male group. (C) ROC of CHA2DS2‐VASc score for the prediction of MACEs in the female group.

In all subjects, the AUC of CHA_2_DS_2_‐VASc score for the prediction of MACE was 0.661 with 74.1% sensitivity and 50.4% specificity (*p* < .001). ROC of LVEF and creatinine for the prediction of MACE was also made, with 0.578 of AUC for LVEF (82.6% sensitivity and 32.2% specificity (*p* < .05)), and 0.571 of AUC for creatinine (27.3% sensitivity and 86.1% specificity (*p* < .05) (Figure 2A). The difference of AUC between CHA_2_DS_2_‐VASc score and LVEF in predicting MACE is statistically significant (*Z* = 2.257, *p* < .05), and also between CHA_2_DS_2_‐VASc score and creatinine (*Z* = 2.503, *p* < .05). For subgroup analysis, the AUC of CHA_2_DS_2_‐VASc score for the prediction of MACE was 0.714 (69.4% sensitivity and 63.1% specificity) (*p* < .001) in the male group (Figure 2B), but there was no statistical significance in the female group (Figure 2C), detailed in Supporting Information: Table 6.

## 4 DISCUSSION

The main finding of this study is that the CHA_2_DS_2_‐VASc scoring system plays an extra role in the prediction of in‐hospital MACE in STEMI patients undergoing pPCI. High scores were independent predictors of total MACE and may be useful for risk stratification. The results of the ROC curve analysis indicated that the CHA_2_DS_2_‐VASc score had moderate predictive efficiency for predicting in‐hospital MACE and the predictive value of the CHA_2_DS_2_‐VASc score for MACE is superior to LVEF and creatinine. Moreover, for subgroup analysis, our data uncovered that the CHA_2_DS_2_‐VASc score is associated with the incidence of in‐hospital MACE in male patients, but not in the female.

Previously, the CHA_2_DS_2_‐VASc score was used in the clinic to assess the risk of thromboembolism in AF patients and guide anticoagulation treatment.^5^ A study found that a higher CHA_2_DS_2_‐VASc score was associated with a significant increase in 1‐year mortality in patients with ACS.^7^ They also found that patients with a CHA_2_DS_2_‐VASc score > 5 had the highest 1‐year mortality risk, sixfold higher compared to patients with a score of 0–1.^7^ Another study by Akboga et al.^9^ demonstrated that CHA_2_DS_2_‐VASc score was independently associated with a higher risk of in‐hospital mortality in NSTEMI patients without AF in a multiple Cox‐regression model. Chen et al.^11^ found that the CHA_2_DS_2_‐VASc score was correlated with the 1‐year major adverse cardiocerebral vascular event in 29 452 AMI patients who were discharged alive. Additionally, studies illustrated that CHA_2_DS_2_‐VASc scores were significantly associated with hospitalization time and adverse events during hospitalization in STEMI patients.^8^, ^12^ In this study, we found that the CHA_2_DS_2_‐VASc score was an independent predictor for in‐hospital MACE in STEMI patients undergoing pPCI, which is in agreement with previous studies. Our study further elucidated that there is a gender‐related difference in CHA_2_DS_2_‐VASc score in predicting MACE.

The gender‐related difference has been demonstrated to exist in the assessment, treatment, and outcomes of CAD,^13^, ^14^ and it has been a hot area of investigation in the past few years. Clinically, female patients are more prone to have atypical symptoms such as pain in the jaw, throat, neck, shoulder, arm, hand, and back, mild pain, and nausea rather than typical chest pain.^15^ Moreover, females are older and have more coronary risk factors such as hypertension, diabetes, and stroke than males.^16^, ^17^, ^18^ Also, women presenting with AMI had a lower likelihood of receiving guideline‐based AMI therapies compared with men.^19^, ^20^ Previous studies demonstrated that females have a higher incidence of MACE compared with males,^21^, ^22^ which is in accordance with our study. Other studies got the opposite results. You et al.^15^ uncovered that the incidences of in‐hospital MACE showed no significant gender difference in 337 elderly patients with STEMI who underwent pPCI. However, the cumulative MACE showed a significant gender‐related difference in the 12‐month follow‐up. We speculate that may be induced by the age difference, racial differences, and also the difference in the follow‐up period. In our study, gender was a predictor of MACE in univariate logistic regression analysis, but not an independent predictor of MACE after multivariate logistic regression analysis. Thus, further studies with larger samples and longer study duration should be conducted to elucidate these relationships.

Kidney disease is associated with an increased risk of death and cardiovascular events in patients with a broad range of cardiovascular diseases, including HF^23^, ^24^, ^25^ and ACS.^26^ Creatinine has been well established to be a risk factor for prognosis in patients with AMI. Michael et al.^27^ found that elevated creatinine and/or reduced creatinine clearance on presentation is associated with increased mortality independent of other conventional risk factors in STEMI patients with mildly or severely impaired renal function. Findings from 14 527 participants in the Valsartan in Acute Myocardial Infarction Trial revealed that mild renal disease should be considered a major risk factor for cardiovascular complications after myocardial infarction.^28^ Moreover, Xu et al.^26^ investigated that mild renal injury has a higher attributable risk to MACE during hospitalization in the Chinese ACS population compared with moderate to severe renal injury. Another study enrolled 11 390 AMI patients without any cardiovascular risk factors and found that serum creatinine levels were an independent predictor of 1‐year MACE.^29^ However, the results of the TRAndopril Cardiac Evaluation register study showed that only severely reduced renal function is associated with an important and independent risk of mortality after AMI.^30^ In agreement with previous studies, our study showed that creatinine was independently correlated with short‐term in‐hospital MACE. Moreover, we did a subgroup analysis and found that creatinine was also positively correlated with short‐term in‐hospital MACE in the male group and female group. Further studies to illustrate the difference between mild, moderate, and severe kidney injury on MACE are warranted.

## 5 LIMITATION

There are several limitations to our study. First, all the data of this study came from only one center and a small sample size, multicenter studies including a greater sample size may be needed in the future. Second, mechanism research was lacking in this study, and further research to fully understand the mechanism behind the association between CHA_2_DS_2_‐VASc score and the prognosis of patients who suffered from AMI undergoing pPCI is needed. Third, this is a retrospective study, we hope further prospective study should be made in the future.

## 6 CONCLUSION

In conclusion, this study indicated that the CHA_2_DS_2_‐VASc scoring system plays an extra role in the prediction of in‐hospital MACE in STEMI patients undergoing pPCI. High scores were an independent predictor of in‐hospital MACE and may be useful for risk stratification. Furthermore, for subgroup analysis, our data uncovered that the CHA_2_DS_2_‐VASc score is associated with the incidence of MACE in male patients, but not in the female group.

## CONFLICT OF INTEREST STATEMENT

The authors declare no conflict of interest.

## Supporting information

Supporting information.

Supporting information.

Supporting information.

Supporting information.

Supporting information.

Supporting information.

## Data Availability

Original research data will be available upon reasonable request.
