# Supplementary material for: Predictive value of CHA2DS2‐VASc score for in‐hospital prognosis of patients with acute ST‐segment elevation myocardial infarction undergoing primary PCI
Source: Clin Cardiol. 2023 Jul 10;46(8):950–7. doi: 10.1002/clc.24071 (PMC10436800; doi:10.1002/clc.24071)
Supplement: Supplementary file 1 — Supporting information. [file CLC-46-950-s002.doc]

Supplementary Table 1. Basic characteristics of patients with CHA_2_DS_2_-VAS_C_ Score difference.

| Variable | CHA_2_DS_2_-VAS_C_ Score Category | | P-value |
| --- | --- | --- | --- |
|  | Low < 2, n = 124 | High ≥ 2, n = 622 |  |
| Age (year) | 51 (14) | 64 (16) | < 0.001 |
| Smoking, n (%) | 92 (74.2) | 313 (50.3) | < 0.001 |
| DM, n (%) | 0 (0) | 198 (100) | < 0.001 |
| Hypertension, n (%) | 0 (0) | 396 (63.7) | < 0.001 |
| Family history, n (%) | 4 (3.2) | 80 (12.9) | 0.002 |
| Past CAD, n (%) | 11 (8.9) | 41 (6.6) | 0.363 |
| Male, n (%) | 124 (100) | 435 (69.9) | < 0.001 |
| Time (h) | 3.0 (4.0) | 4.0 (4.0) | 0.186 |
| Heart rate (bpm) | 78.32 ± 17.03 | 76 (20) | 0.265 |
| Hemoglobin (g/L) | 149 (14) | 141.50 (22) | < 0.001 |
| WBC count (×10^9^/L) | 10.28 (3.96) | 9.67 (4.06) | 0.002 |
| NEU (×10^9^/L) | 7.93 (3.87) | 7.43 (4.12) | 0.054 |
| PLT (×10^9^/L) | 234 (77) | 227 (79) | 0.253 |
| LYM (×10^9^/L) | 1.39 (1.12) | 1.34 (0.96) | 0.342 |
| Cr (umol/L) | 66.45 (17) | 62.10 (22) | 0.033 |
| TC (mmol/L) | 4.80 (1.17) | 4.67 (1.35) | 0.349 |
| TG (mmol/L) | 1.49 (1.18) | 1.46 (1.24) | 0.637 |
| LVEF (%) | 50.52 ± 7.04 | 50 (10) | 0.377 |
| Fib (ng/ml) | 2.92 (0.75) | 3.02 (0.89) | 0.021 |
| D-dimer (ng/ml) | 0.30 (0.40) | 0.33(0.51) | 0.181 |
| N/L | 6.33 (6.22) | 5.79 (5.57) | 0.489 |

**Abbreviation:** DM: diabetes mellitus; CAD: coronary artery disease; WBC: white blood cell; NEU: neutrophils; PLT: platelet; LYM: lymphocyte; Cr: creatinine; TC: total cholesterol; TG: triglyceride; LVEF: left ventricular ejection fraction; Fib: fibrinogen; N/L: neutrophils to lymphocyte ratio.
