# Supplementary material for: Predictive value of CHA2DS2‐VASc score for in‐hospital prognosis of patients with acute ST‐segment elevation myocardial infarction undergoing primary PCI
Source: Clin Cardiol. 2023 Jul 10;46(8):950–7. doi: 10.1002/clc.24071 (PMC10436800; doi:10.1002/clc.24071)
Supplement: Supplementary file 2 — Supporting information. [file CLC-46-950-s004.doc]

Supplementary Table 2. Basic characteristics of male patients with CHA2DS2-VASC Score difference.

| Characteristics | 1 | 2-3 | 4-5 | ﹥5 | p-value |
| --- | --- | --- | --- | --- | --- |
|  | n = 124 | n = 313 | n = 102 | n = 20 |  |
| Age (year) | 51 (14) | 59 (16) | 66.5 (14) | 72.45 ± 5.45 | < 0.001 |
| Smoking, n (%) | 92 (74.2) | 216 (69) | 70 (68.6) | 16 (80) | 0.535 |
| DM, n (%) | 0 | 87 (27.8) | 34 (33.3) | 15 (75) | < 0.001 |
| Hypertension, n (%) | 0 | 203 (64.9) | 69 (67.6) | 17 (85) | < 0.001 |
| CAD, n (%) | 4 (3.2) | 33 (10.5) | 17 (16.7) | 5 (25) | 0.001 |
| History, n (%) | 11 (8.9) | 28 (8.9) | 10 (9.8) | 0 (0) | 0.282 |
| Time (h) | 3 (4) | 3.5 (4) | 3 (4) | 5.25 (5.13) | < 0.05 |
| Heart rate (bpm) | 78.3 ± 17.10 | 76 (19) | 73 (22) | 76.50 ± 16.39 | 0.142 |
| Hemoglobin (g/L) | 149 (14) | 149 (19) | 141.97 ± 17.25 | 136.75± 12.10 | < 0.001 |
| WBC count (×10^9^/L) | 10.29 (3.97) | 9.96 (4.22) | 8.94 (4.25) | 10.64 ± 3.97 | ＜0.05 |
| NEU (×10^9^/L) | 8.0 (3.88) | 7.54 (4.35) | 7.03 (3.96) | 8.74 ± 3.97 | 0.144 |
| PLT (×10^9^/L) | 234 (78) | 229 (78) | 206.5 (63) | 218.3 ± 46.5 | 0.189 |
| LYM (×10^9^/L) | 1.40 (1.10) | 1.42 (1.02) | 1.29 (0.92) | 1.29 (0.70) | 0.125 |
| Creatinine (umol/L) | 66 (17) | 64.70 (19) | 68.65 (18) | 73.05 (36) | < 0.05 |
| TC (mmol/L) | 4.80 (1.18) | 4.62 (1.19) | 4.38 (1.13) | 4.32 ± 1.45 | < 0.05 |
| TG (mmol/L) | 1.49 (1.18) | 1.46 (1.40) | 1.47 (1.13) | 1.57 ± 0.85 | 0.931 |
| LVEF (%) | 50.00 ± 7.06 | 50 (9) | 51 (9) | 50.50 ± 7.89 | 0.596 |
| Fib (ng/ml) | 2.92 (0.75) | 2.98 (0.94) | 3.06 (0.92) | 2.95 ± 0.77 | 0.177 |
| D-dimer (ng/ml) | 0.30 (0.41) | 0.29 (0.45) | 0.40 (0.52) | 0.48 (0.71) | < 0.05 |
| N/L | 6.17 (6.18) | 5.50 (5.59) | 6.41 (5.61) | 7.50 (6.47) | 0.413 |

**Abbreviation:** DM: diabetes mellitus; CAD: coronary artery disease; WBC: white blood cell; NEU: neutrophils; PLT: platelet; LYM: lymphocyte; TC: total cholesterol; TG: triglyceride; LVEF: left ventricular ejection fraction; Fib: fibrinogen; N/L: neutrophils to lymphocyte ratio.
