# Supplementary material for: Predictive value of CHA2DS2‐VASc score for in‐hospital prognosis of patients with acute ST‐segment elevation myocardial infarction undergoing primary PCI
Source: Clin Cardiol. 2023 Jul 10;46(8):950–7. doi: 10.1002/clc.24071 (PMC10436800; doi:10.1002/clc.24071)
Supplement: Supplementary file 3 — Supporting information. [file CLC-46-950-s006.doc]

Supplementary Table 3. Basic characteristics of female patients with CHA2DS2-VASC Score difference.

| Characteristics | 1 | 2-3 | 4-5 | ﹥5 | p-value |
| --- | --- | --- | --- | --- | --- |
|  | n = 0 | n = 78 | n = 83 | n = 26 |  |
| Age (year) |  | 63 (8) | 69 (9) | 72.38 ± 7.60 | < 0.001 |
| Smoking, n (%) |  | 5 (6.4) | 6 (7.2) | 0 | 0.264 |
| DM, n (%) |  | 13 (16.7) | 35 (42.2) | 14 (53.8) | < 0.001 |
| Hypertension, n (%) |  | 28 (35.9) | 58 (69.9) | 21 (80.5) | < 0.001 |
| CAD, n (%) |  | 9 (11.5) | 10 (12) | 6 (23.1) | 0.221 |
| History, n (%) |  | 2 (2.6) | 1 (1.2) | 0 (0) | 0.517 |
| Time (h) |  | 4 (3.5) | 4 (3) | 5.38 ± 2.74 | 0.164 |
| Heart rate (bpm) |  | 74.94 ± 14.40 | 75 (20) | 78.5 (21) | 0.497 |
| Hemoglobin (g/L) |  | 130.5 (19) | 131 (21) | 129.58± 11.46 | 0.843 |
| WBC count (×10^9^/L) |  | 9.4 ± 2.67 | 9.81 (3.19) | 10.12 ± 3.33 | 0.501 |
| NEU (×10^9^/L) |  | 7.6 ± 2.8 | 7.8 ± 2.83 | 8 ± 3.16 | 0.798 |
| PLT (×10^9^/L) |  | 242 (95) | 235.77 ± 50.55 | 261 (92) | 0.668 |
| LYM (×10^9^/L) |  | 1.19 (0.83) | 1.36 (0.88) | 1.35 (0.86) | 0.121 |
| Creatinine (umol/L) |  | 50.15 (17) | 55.5 (18) | 56.23 ± 15.97 | 0.022 |
| TC (mmol/L) |  | 5.19 ± 1.13 | 4.98 ± 0.95 | 5.28 ± 0.94 | 0.282 |
| TG (mmol/L) |  | 1.40 (1.02) | 1.40 (1.25) | 1.58 (1.20) | 0.403 |
| LVEF (%) |  | 50.78 ± 7.67 | 50.13 ± 8.72 | 51.31 ± 6.24 | 0.769 |
| Fib (ng/ml) |  | 2.99 (0.82) | 3.04 (0.76) | 3.21 ± 0.71 | 0.379 |
| D-dimer (ng/ml) |  | 0.40 (0.47) | 0.50 (0.66) | 0.44 (0.69) | 0.248 |
| N/L |  | 6.48 (6.30) | 5.63 (4.99) | 4.97 (5.88) | 0.417 |

**Abbreviation:** DM: diabetes mellitus; CAD: coronary artery disease; WBC: white blood cell; NEU: neutrophils; PLT: platelet; LYM: lymphocyte; TC: total cholesterol; TG: triglyceride; LVEF: left ventricular ejection fraction; Fib: fibrinogen; N/L: neutrophils to lymphocyte ratio.
