# Supplementary material for: Predictive value of CHA2DS2‐VASc score for in‐hospital prognosis of patients with acute ST‐segment elevation myocardial infarction undergoing primary PCI
Source: Clin Cardiol. 2023 Jul 10;46(8):950–7. doi: 10.1002/clc.24071 (PMC10436800; doi:10.1002/clc.24071)
Supplement: Supplementary file 4 — Supporting information. [file CLC-46-950-s005.doc]

Supplementary Table 4. Logistic regression analysis to show MACE predicted by CHA_2_DS_2_-VAS_C_ score in male patients.

| Scoring algorithm | Univariable analysis | | |  | Multivariable analysis | | |
| --- | --- | --- | --- | --- | --- | --- | --- |
|  | OR | 95%CI | p |  | AOR | 95%CI | p |
| CHA_2_DS_2_-VAS_C_ (continuous variable) ^*^ | 1.74 | 1.48-2.05 | < 0.001 |  | 1.71 | 1.44-2.02 | < 0.001 |
| CHA_2_DS_2_-VAS_C_ (category variable) ^†^ |  |  |  |  |  |  |  |
| 1 | Reference | - | - |  | Reference | - | - |
| 2-3 | 3.83 | 1.60-9.17 | 0.003 |  | 3.76 | 1.56-9.08 | 0.003 |
| 4-5 | 7.81 | 3.09-19.73 | < 0.001 |  | 7.46 | 2.91-19.10 | < 0.001 |
| > 5 | 29.50 | 8.76-99.30 | < 0.001 |  | 26.31 | 7.50-92.24 | < 0.001 |

**Abbreviation:** MACE: major adverse cardiovascular event; OR: odds ratio; CI: confidence interval; AOR: adjusted odds ratio. *The multivariable analysis included the CHA_2_DS_2_-VASc score as a continuous variable, creatinine, total cholesterol; left ventricular ejection fraction. ^†^The multivariable analysis included the CHA2DS2-VASc score as a category variable, creatinine, total cholesterol; left ventricular ejection fraction.
