# Supplementary material for: Predictive value of CHA2DS2‐VASc score for in‐hospital prognosis of patients with acute ST‐segment elevation myocardial infarction undergoing primary PCI
Source: Clin Cardiol. 2023 Jul 10;46(8):950–7. doi: 10.1002/clc.24071 (PMC10436800; doi:10.1002/clc.24071)
Supplement: Supplementary file 5 — Supporting information. [file CLC-46-950-s003.doc]

Supplementary Table 5. Logistic regression analysis to show MACE predicted by CHA_2_DS_2_-VAS_C_ score in female patients.

| Scoring algorithm | Univariable analysis | | |  | Multivariable analysis | | |
| --- | --- | --- | --- | --- | --- | --- | --- |
|  | OR | 95%CI | p |  | AOR | 95%CI | p |
| CHA_2_DS_2_-VAS_C_ (continuous variable) ^*^ | 0.879 | 0.682-1.133 | 0.320 |  | 0.84 | 0.64-1.10 | 0.207 |
| CHA_2_DS_2_-VAS_C_ (category variable) ^†^ |  |  |  |  |  |  |  |
| 2-3 | Reference | - | - |  | Reference | - | - |
| 4-5 | 0.98 | 0.48-1.99 | 0.961 |  | 0.79 | 0.38-1.69 | 0.552 |
| > 5 | 0.53 | 0.162-1.72 | 0.288 |  | 0.47 | 0.14-1.61 | 0.23 |

**Abbreviation:** MACE: major adverse cardiovascular event; OR: odds ratio; CI: confidence interval; AOR: adjusted odds ratio. *The multivariable analysis included the CHA_2_DS_2_-VASc score as a continuous variable, creatinine, left ventricular ejection fraction. ^†^The multivariable analysis included the CHA_2_DS_2_-VASc score as a category variable, creatinine, left ventricular ejection fraction.
