# Supplementary material for: Predictive value of CHA2DS2‐VASc score for in‐hospital prognosis of patients with acute ST‐segment elevation myocardial infarction undergoing primary PCI
Source: Clin Cardiol. 2023 Jul 10;46(8):950–7. doi: 10.1002/clc.24071 (PMC10436800; doi:10.1002/clc.24071)
Supplement: Supplementary file 6 — Supporting information. [file CLC-46-950-s001.doc]

Supplementary Table 6. Performance of variables in predicting short-term major adverse cardiac event.

| Variable | AUC | Sensitivity | Specificity | p-value |
| --- | --- | --- | --- | --- |
| CHA_2_DS_2_-VASc | 0.661 | 74.1 | 50.4 | < 0.001 |
| LVEF | 0.578 | 82.6 | 32.2 | < 0.05 |
| Creatinine | 0.571 | 27.3 | 86.1 | < 0.05 |
| Male CHA_2_DS_2_-VASc | 0.714 | 69.4 | 63.1 | < 0.001 |
| Female CHA_2_DS_2_-VASc | 0.542 | - | - | 0.392 |

**Abbreviation:** AUC: area under curve; LVEF: left ventricular ejection fraction.
